# Survival Outcomes and Health-Related Quality of Life in Older Adults Diagnosed with Acute Myeloid Leukemia Receiving Frontline Therapy in Daily Practice

**DOI:** 10.3390/jpm13121667

**Published:** 2023-11-28

**Authors:** Fernando Ramos, María Lourdes Hermosín, Marta Fuertes-Núñez, Pilar Martínez, Carlos Rodriguez-Medina, Manuel Barrios, Francisco Ibáñez, Teresa Bernal, Maria Teresa Olave, Miguel Ángel Álvarez, María Vahí, Teresa Caballero-Velázquez, Bernardo González, Albert Altés, Lorena García, Pascual Fernández, María Antonia Durán, Rocío López, Montserrat Rafel, Josefina Serrano

**Affiliations:** 1 Department of Hematology, Hospital Universitario de León, 24008 Leon, Spain; 2 Department of Hematology, Hospital Universitario de Jerez de la Frontera, 11407 Jerez de la Frontera, Spain; 3 Department of Hematology, Hospital Universitario 12 de Octubre, 28041 Madrid, Spain; 4 Department of Hematology, Hospital Universitario de Gran Canaria Dr. Negrín, 35010 Las Palmas de Gran Canaria, Spain; 5 Department of Hematology, Hospital Regional Universitario de Málaga, 29010 Malaga, Spain; 6 Department of Hematology, Hospital General Universitario de Valencia, 46014 Valencia, Spain; 7 Department of Hematology, Hospital Universitario Central de Asturias, 33011 Oviedo, Spain; 8 Department of Hematology, Hospital Clinico Lozano Blesa, 50009 Zaragoza, Spain; 9 Department of Hematology, Hospital Universitario de Getafe, 28905 Getafe, Spain; 10 Department of Hematology, Hospital Universitario Virgen de Valme, 41014 Sevilla, Spain; 11 Department of Hematology, Instituto de Biomedicina de Sevilla, IBiS/Hospital Universitario Virgen del Rocío/CSIC/Universidad de Sevilla, 41013 Sevilla, Spain; 12 Department of Hematology, Hospital Universitario de Canarias, 38320 La Laguna, Spain; 13 Department of Hematology, Hospital Sant Joan de Deu de Manresa—Fundació Althaia, 08243 Manresa, Spain; 14 Department of Hematology, Complejo Hospitalario Universitario A Coruña (CHUAC), 15006 A Coruña, Spain; 15 Department of Hematology, Hospital General Universitario de Alicante, 03010 Alicante, Spain; 16 Department of Hematology, Hospital Universitario Son Espases, 07120 Palma de Mallorca, Spain; 17 Medical Department, Hematology Area, Bristol Myers Squibb Company, Celgene, 28050 Madrid, Spain; 18 Department of Hematology, Hospital Universitario Reina Sofía, IMIBIC UCO, 14004 Cordoba, Spain

**Keywords:** acute myeloid leukemia, elderly, survival, early death, health-related quality of life, life expectancy, geriatric assessment, fatigue

## Abstract

Acute myeloid leukemia has a poor prognosis in older adults, and its management is often unclear due to its underrepresentation in clinical trials. Both overall survival (OS) and health-related quality-of-life (HRQoL) are key outcomes in this population, and patient-reported outcomes may contribute to patient stratification and treatment assignment. This prospective study included 138 consecutive patients treated in daily practice with the currently available non-targeted therapies (intensive chemotherapy [IC], attenuated chemotherapy [AC], hypomethylating agents [HMA], or palliative care [PC]). We evaluated patients’ condition at diagnosis (Life expectancy [Lee Index for Older Adults], Geriatric Assessment in Hematology [GAH scale], HRQoL [EQ-5D-5L questionnaire], and fatigue [fatigue items of the QLQ-C30 scale]), OS, early death (ED), treatment tolerability (TT) and change in HRQoL over 12 months follow-up. The median OS was 7.1 months (IC not reached, AC 5.9, HMA 8.8, and PC 1.0). Poor risk AML category and receiving just palliative care, as well as a higher Lee index score in the patients receiving active therapy, independently predicted a shorter OS. The Lee Index and GAH scale were not useful for predicting TT. The white blood cell count was a valid predictor for ED. Patients’ HRQoL remained stable during follow-up.

## 1. Introduction

Acute myeloid leukemia (AML) is the most commonly diagnosed type of acute leukemia [1] and occurs mainly in patients aged over 60 years. There are large differences between older and younger patients in clinical prognosis, with higher rates of early death (ED) and chemotherapy-related toxicities in the former [2,3]. The prognosis for older adults with AML can be estimated with the Medical Research Council/Leukemia Research Foundation (MRC/LRF), the MD Anderson Cancer Center, and the European LeukemiaNet prognostic scores [4,5,6], among others.

Despite the higher prevalence of this hematologic cancer in the elderly, its management is still unclear in this patient population [7]. Fit older adults treated by intensive chemotherapy (IC), especially if they are included in the good risk category or followed by either allogeneic hematopoietic cell transplantation (HCT) or oral azacitidine maintenance, have prolonged their overall survival (OS) [8,9,10,11]. Nevertheless, many older patients have worse health status (poor performance status, multimorbidity, polypharmacy, malnutrition, cognitive impairment, etc.) and may have difficulties in following the above-mentioned clinical pathway and have reduced survival [11,12]. The available options for old and unfit patients are hypomethylating agents (HMA), such as azacitidine or decitabine [13,14,15,16], attenuated standard chemotherapy (AC), and palliative care (PC). Recently, the combination of azacitidine and venetoclax has shown improved overall survival, as compared to azacitidine plus placebo, in newly diagnosed unfit patients [17].

It is crucial to evaluate patient characteristics and disease heterogeneity thoroughly to offer appropriate personalized therapy [18,19]. Currently, patient evaluation is mainly based on traditional factors, including age, Eastern Cooperative Oncology Group (ECOG) performance status, and comorbidity, but this approach does not fully describe these patients [20]. Patient-reported outcomes (PROs) such as health-related quality-of-life (HRQoL), fatigue perception, etc., are currently gaining relevance in treatment decision-making. Studying the impact of both traditional factors and PROs in a multidimensional evaluation will help us to understand the reasons underlying treatment assignment and guide the choice of the most appropriate therapy. A prominent unmet clinical need is the lack of straightforward tools to predict early death (ED) and treatment tolerability (TT) in patients receiving the different forms of therapy considered since most available information comes from the IC setting [3,5,12,15,21,22,23,24,25].

In light of the above, we aimed to describe the OS of older adults diagnosed with AML and the change in their HRQoL as a function of the treatment strategy: to analyze the relative prognostic value for OS of life expectancy, geriatric assessment, fatigue, and HRQoL at diagnosis, adjusted for MRC/LRF risk categories; to study in depth the impact of white blood cell count (WBC) and other covariates on ED and TT; and, lastly, to describe the transfusion burden and the need for hospitalization for each treatment strategy, as well as the hematopoietic cell transplantation (HCT) rate.

## 2. Materials and Methods

### 2.1. Study Design and Participants

SVLMA was a prospective observational study carried out by the Departments of Hematology of 40 Spanish hospitals according to the Helsinki Declaration and local regulations. It was approved by the Independent Ethics Committee of Hospital Clínico San Carlos (Madrid, Spain), and all patients gave their written informed consent.

This study included all patients meeting the selection criteria who were consecutively recruited from February 2018 to April 2019. Eligible patients were aged ≥60 years and diagnosed with AML as defined in the World Health Organization (WHO) 2016 criteria [26]. Patients with previously treated AML or acute promyelocytic leukemia were excluded.

Included patients were treated with all strategies available at the outset of the study in clinical practice: IC, AC, HMA, or PC. IC comprised any “3 + 7” scheme including cytarabine daily doses 100 mg/sqm and over, in combination with either (i) daunorubicin at a daily dose of at least 60 mg/sqm (or equivalent anthracycline dose) or (ii) fludarabine (any dose), and the FLAG-IDA scheme. Lower doses of the same drugs or shorter schedules were considered AC. Each therapy was administered at the discretion of the participating physician and according to routine clinical practice.

### 2.2. Assessments and Endpoints

Patients were followed up for 12 months and assessed at 5 timepoints: baseline/selection visit and four consecutive visits every 3 months (at 0, 3, 6, 9, and 12 months).

The primary study endpoint was the median OS, defined as the time from diagnosis to death from any cause. Secondary efficacy endpoints included 1-year survival rate, treatment strategies, ED, TT, and change in patient HRQoL. We also studied the potential impact of patient conditions (life expectancy, performance status, geriatric assessment, and fatigue scores) on ED and TT, as well as the potential impact of WBC at diagnosis on ED. Additionally, data about treatment response, transfusion burden, need for hospitalization, HCT rate, and the date and reason of death were also collected.

HRQoL was measured by the EuroQoL-5L-5D questionnaire [27], and fatigue was scored (FA-score) by using the 3 fatigue items of the QLQ-C30 [28]. Life expectancy (at 4 years) before AML diagnosis was calculated with the Lee Index for Older Adults [29]. Geriatric assessment was performed with the aid of the Geriatric Assessment in Hematology (GAH) scale [30,31]. Details on these scales can be consulted in the Supplementary Materials.

### 2.3. Statistical Methods

Median OS and 1-year survival rate were analyzed using the Kaplan-Meier method. Those patients receiving HCT were censored. A Cox regression multivariate analysis was used to determine the impact of the covariates to predict OS. Variables showing significant differences (*p* < 0.150) in the univariate analyses were included in a multivariate analysis. 

ED was defined as the patient’s death within the first 8 weeks of treatment initiation [32]. 

TT was measured as the number of patients that needed either treatment termination or modification (i.e., at the patient’s request, when the patient had any adverse event that was considered severe or life-threatening by the investigator, and also when the patient experienced any deterioration of previous comorbidities or early death as a consequence of the adverse event). In addition, the physician’s subjective opinion on the observed patient tolerability on a scale of 0 (minimal) to 10 (maximal) was also collected.

Treatment response was defined following the European LeukemiaNet criteria [6]. 

Transfusion burden was calculated as the number of packed red blood cell concentrates (PRBC) and the number of platelet transfusion procedures received by each patient during follow-up. Need for hospitalization was analyzed as the proportion of patients requiring hospitalization (excluding hospice or palliative care stays) during follow-up, as well as the proportion of days that the patients spent at the hospital as in-patients during the observation time.

The impact of WBC on ED was analyzed by ANCOVA (accounting for time from diagnosis to treatment initiation) and a receiver operating characteristics (ROC) curve. The area under the curve (AUC) was calculated to identify the cut-off point, maximizing sensitivity and specificity. HRQoL was descriptively analyzed, including the frequency distributions of EQ-5D-5L dimensions (mobility, self-care, usual activities, pain/discomfort, and anxiety/depression) and measures of central tendency and dispersion of EQ-VAS and preference indexes; the latter was also analyzed using repeated-measures linear models. The Lee index and GAH scale validity as treatment-tolerability predictive values were analyzed by a full multiple linear regression model and multiple logistic regression. Optimal cut-off points were determined by ROC analysis.

All the variables were analyzed in the overall series and adjusted by treatment. Missing data were not imputed and were left as missing. The hypothesis tests used were two-sided and with a significance level of 0.05. IBM SPSS Statistics version 22.0 (IBM Corp., Armonk, NY, USA) was used for all the analysis.

## 3. Results

### 3.1. Patient Disposition and Frontline Treatment

**Figure 1:**
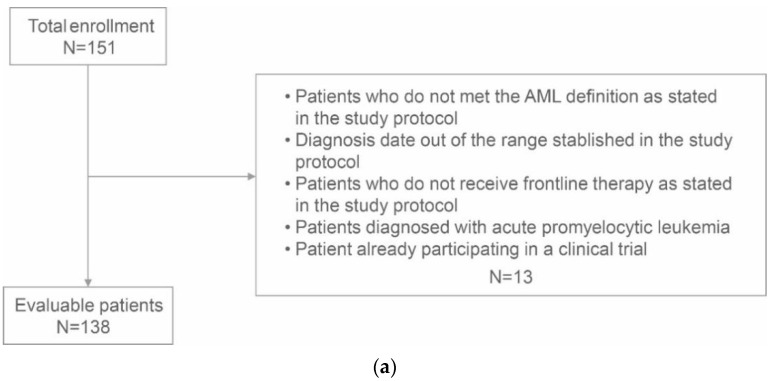

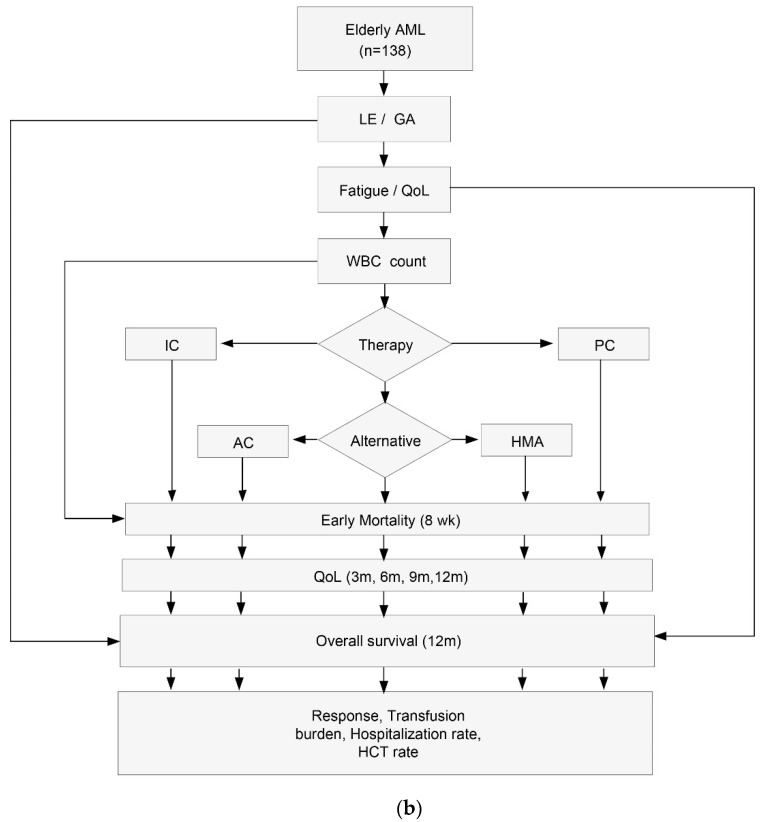
(**a**) Patient enrollment flowchart. (**b**). Study flowchart. AML, acute myeloid leukemia; LE, Life expectancy; GA, Geriatric assessment; QoL, quality-of-life; WBC, white blood cell count; IC, intensive chemotherapy; AC, attenuated chemotherapy; HMA, hypomethylating agents; PC, palliative care; wk, week; m, month; HCT, hematopoietic cell transplantation.

A total of 151 patients were enrolled in the study, 13 of whom did not meet inclusion criteria, so 138 were finally evaluable (Figure 1, panel a). All treatments were administered according to the physician’s criteria and the local clinical practice. The study flow is displayed in Figure 1, panel b. and patient characteristics are shown in Table 1. Median (interquartile range, IQR) patient age was 75.2 (68.6–80.6) years, and most were male (57.2%). Patient ECOG performance status was predominantly good (ECOG 0–1 72.6%), but 27.4% of patients had an ECOG score of 2–3 (Table 1); 35 patients out of 137 (25.5%) had a previous hematological disease, and 27 (19.6%) had previously received chemotherapy and/or radiotherapy.

**Figure 2:**
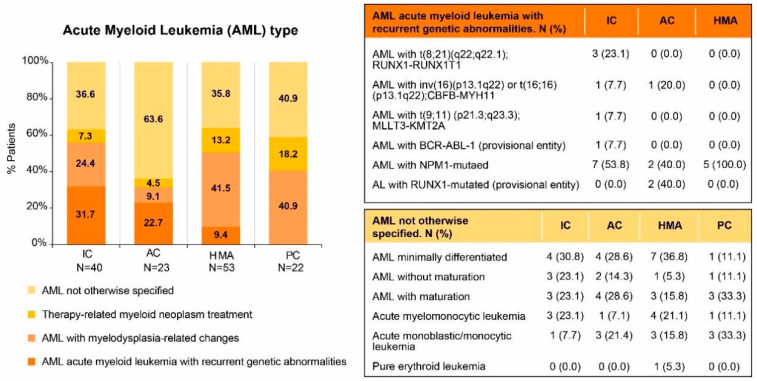
AML type following WHO 2016 classification criteria. AC, attenuated chemotherapy; AML, acute myeloid leukemia; IC, intensive chemotherapy; HMA, hypomethylating agents; PC, palliative care.

Fifty-five patients (39.9%) had an abnormal karyotype, and in 17 (13.2%) it was complex. In one case of the HMA group, cytogenetics was not available. Among the patients with normal cytogenetics, the prevalence of mutations in the genes *NPM1*, *FLT3*, and *CEBPA* was 9.3%, 18.6%, and 3.2%, respectively. Details on WHO classification are displayed in Figure 2 (genomic data available in the Supplementary Materials, Table S1).

**Figure 3:**
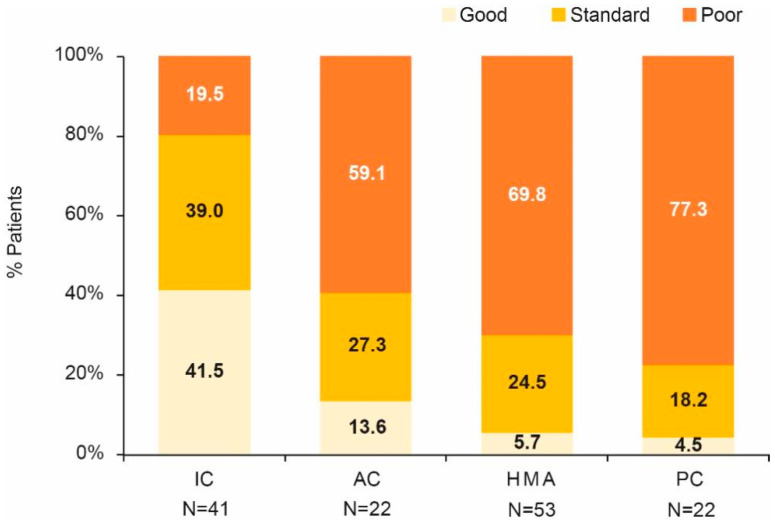
MRC/LRF categories by treatment group. AC, attenuated chemotherapy; IC, intensive chemotherapy; HMA, hypomethylating agents; PC, palliative care.

Most patients (54.3%) were included in the poor MRC/LRF risk category. Good MRC/LRF risk score was predominant in the patients receiving IC, while poor risk score was predominant in the rest of the patients (Figure 3). These differences were statistically significant (*p* < 0.001).

The need for PRBC at diagnosis was maximal for patients who were later assigned to PC and minimal for those assigned IC (Figure S1), but the differences between groups were not statistically significant (*p* = 0.101). 

**Figure 4:**
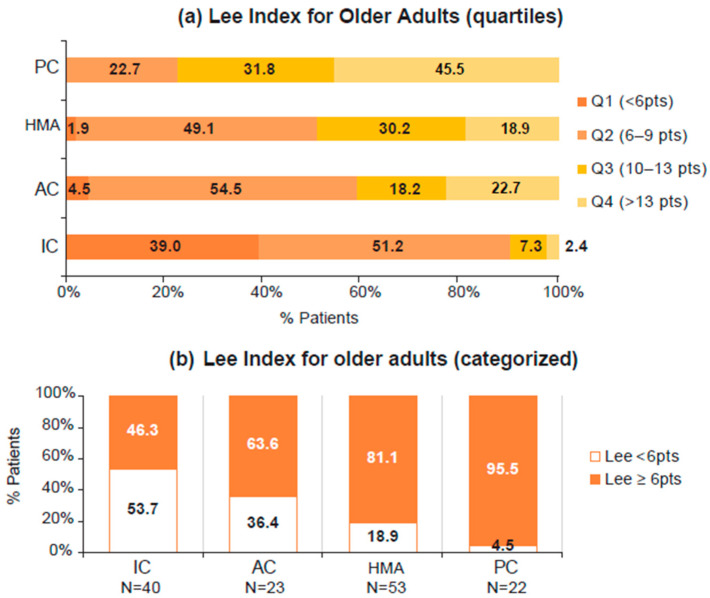
Lee Index for Older Adults by treatment group. (**a**) Distribution of Lee Index Score by quartiles. (**b**). Distribution of Lee Index Score by categories (score < 6 pts and score ≥ 6 pts). AC, attenuated chemotherapy; IC, intensive chemotherapy; HMA, hypomethylating agents; PC, palliative care.

Life expectancy prior to AML diagnosis was maximal in the IC group, minimal in the PC, and intermediate in those treated by AC and HMA (Table 1 and Figure 4). Patient condition, according to the GAH scale, was intermediate and similar across treatment groups (Table 1). Some GAH dimension subscores, more specifically, the number of drugs, difficulties in performing activities of daily life, comorbidities, and smoking habits, were statistically different between the treatment strategies (Table S2). EQ-5D-VAS and fatigue were also similar in the different groups (Table 1; details on the different domains are shown in Table S3).

One hundred and thirty-eight patients initiated frontline therapy according to standard clinical practice (Table S4). The mean time (±SD) from diagnosis to therapy was 9.8 ± 14.3 days. Forty-one patients (29.7%) received IC, 22 (15.9%) AC, 53 (38.4%) HMA, and 22 (15.9%) did not receive active therapy but just PC.

### 3.2. Overall Survival and Prognostic Factors

**Figure 5:**
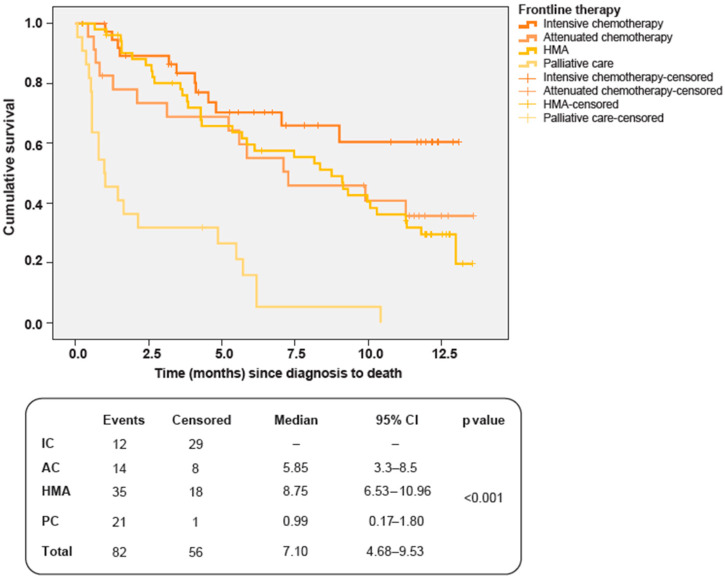
Overall survival by treatment group. AC, attenuated chemotherapy; IC, intensive chemotherapy; HMA, hypomethylating agents; PC, palliative care.

Fifty-six patients (40.6%) died during follow-up. Median OS for the overall series was 7.1 months (95%CI 4.7–9.5), with significant differences by treatment groups: median IC not reached; AC, 5.9 (95% CI 3.3–8.5); HMA, 8.8 (95% CI 6.5–11.0), and PC 1.0 (95% CI 0.2–1.8) (*p* < 0.001) (Figure 5).

Overall, the 1-year survival rate was 31.9% (95% CI 23.1–40.7). When stratified by treatment strategy, 1-year survival was 61.7% (95% CI 43.8–79.6) for patients on IC, 29.8% (95% CI 9.0–50.6) for AC, and 29.5% (95% CI 16.5–42.6) for HMA (Figure S2). Given the absence of data at 12 months follow-up, this variable could not be calculated for patients on PC.

As regards the impact of variables predicting OS, multivariate analysis showed that patients in the MRC/LRF poor category (HR = 2.69, 95% CI = 1.66–4.37, *p* < 0.001) and those receiving PC (HR = 4.97, 95% CI = 2.93–8.43, *p* < 0.001) had a higher mortality risk. No other relevant prognostic factors for OS were identified.

### 3.3. Early Death: Impact of WBC at Diagnosis

Twenty-eight patients (21.5%) died before 8 weeks from diagnosis, fourteen of whom (50%) were receiving PC.

The study hypothesis set out that WBC could be a relevant predictor of ED. When we compared the mean (±SD) WBC of the patients who died early vs. the WBC of those surviving that period, we found higher values for patients with ED (55.0 ± 79.7 × 10^9^/L vs. 19.1 ± 45.7 × 10^9^/L; *p* < 0.001) and a positive correlation between ED proportion and WBC strata (*p* = 0.002, Figures S3 and S4). The ANCOVA analysis, performed from diagnosis to treatment onset, showed similar results (53.0 × 10^9^/L, 95% CI 32.2–73.8 vs. 19.6 × 10^9^/L, 95% CI 8.9–30.4; *p* = 0.006). These results were confirmed by a ROC analysis, demonstrating the validity of WBC for predicting ED in the overall series (AUC = 0.718, 95% CI 0.606–0.829, *p* < 0.001) at the expense of patients treated with alternative chemotherapy (either AC or HMA; AUC = 0.805, 95% CI = 0.698–0.911, *p* = 0.002), but could not be confirmed for those receiving IC (*p* = 0.231). The optimal cut-off was 5.8 × 10^9^ leucocytes/L in the overall series and 9.5 × 10^9^ leucocytes/L in those patients receiving alternative chemotherapy.

### 3.4. Treatment Tolerability

TT, according to the investigator, was considered intermediate: overall (mean ± SD) 6.4 ± 2.9; IC 6.0 ± 2.7; AC 5.5 ± 3.4 and HMA 6.9 ± 2.3. Objective TT was poor in 30/116 (25.9%) of the patients receiving active antileukemic treatment, with no statistically significant differences between the treatment groups (IC 24.4%, AC 22.7%, HMA 28.3%, *p* = 0.85). Reasons for treatment termination and types of treatment modification due to toxicity are shown in Tables S5 and S6.

### 3.5. Treatment Response

The response could not be evaluated in 34 out of 116 (29.3%) patients receiving active treatment, mainly because of the lack of longitudinal bone marrow data. The overall response rate was 54.3% (45/81). By treatment groups, IC-treated patients had a higher response rate than those receiving alternative chemotherapy (IC 28/34, 82.4%; AC or HMA 16/47, 34.0%; *p* < 0.001).

### 3.6. Patient Condition and Patient-Reported Outcomes

Neither Lee index/GAH scores nor fatigue score at diagnosis/EQ-VAS showed independent prognostic value on OS in the overall series (Table 2). When we restricted the OS analysis to those patients receiving active therapy (i.e., excluding PC), MRC/LRF poor category (HR = 2.02, 95% CI = 1.11–3.64, *p* = 0.020) and a higher Lee score (HR = 1.08, 95%CI = 1.002–1.156, *p* = 0.044) were the only covariates linked to a higher mortality risk.

Lee and GAH scores were not associated with ED (*p* = 0.142 and *p* = 0.310, respectively), and as regards TT, neither of them can be considered appropriate tools for predicting TT (AUC 0.429, 95% CI 0.311–0.547, *p* = 0.222 and AUC 0.684, 95% CI 0.521–0.847, *p* = 0.140; respectively).

**Figure 6:**
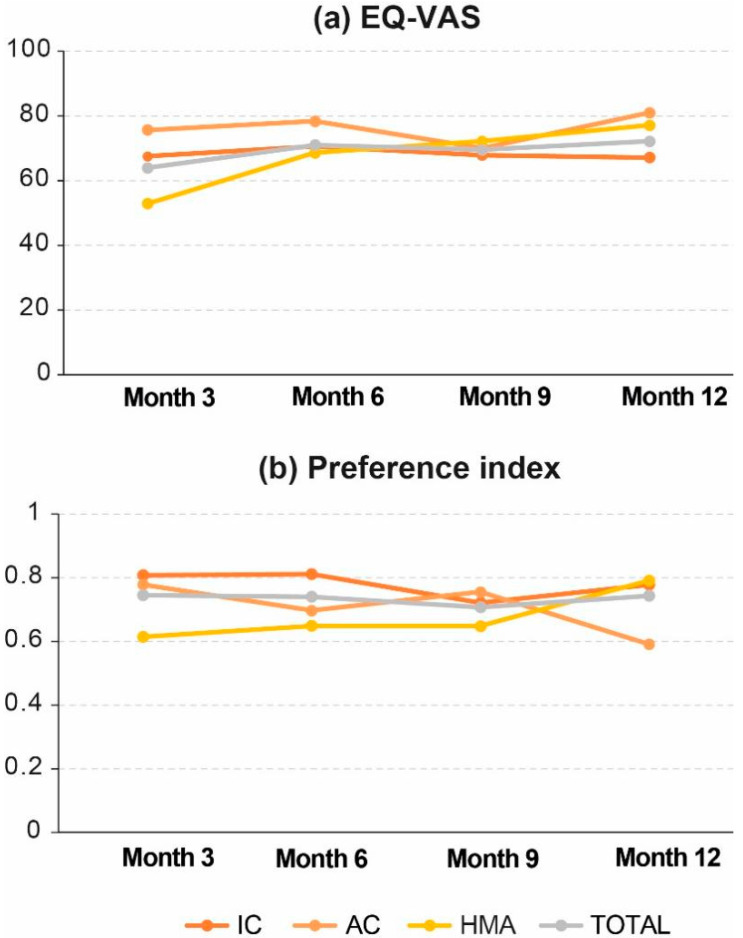
Change in HRQoL during follow-up: (**a**) EQ-VAS and (**b**) Preference index. AC, attenuated chemotherapy; IC, intensive chemotherapy; HMA, hypomethylating agents; PC, palliative care.

Most patients were allocated to the most favorable category of each EQ-5D dimension, and this status was maintained until the end of the study (Figure 6). The score obtained in the preference index was stable during the study follow-up and closer to 1 (perfect health) than to 0 (death). From month 6 onwards, data from patients on PC care were no longer available due to the high mortality rate, so they were not included in the comparative analysis. After 12 months of follow-up, EQ-VAS results indicated a significant improvement in HRQoL in the overall population (*p* = 0.016), and specifically in patients on HMA treatment (*p* = 0.040), but the preference index was not statistically different in either of them (*p* = 0.66, and *p* = 0.08, respectively; Table 3).

### 3.7. Transfusion Burden, Hospitalization and Transplantation

Most patients (71.7%) required PRBC transfusion during the study follow-up. We did not observe statistically significant differences in the number of PRBC transfused between the treatment strategies. However, for the first 3 months of study follow-up, the number of PRBC (mean ± SD) transfused was maximal in IC (15.2 ± 10.1) and AC (16.4 ± 10.4) groups, minimal in the PC subset (9.5 ± 7.6) and intermediate in those patients treated by HMA group (10.6 ± 7.3) (*p* = 0.045).

Concerning platelet transfusions, they were needed by 56.5% of the patients and followed the same trend observed for the PRBC (14.5 ± 11.0, 11.7 ± 9.1, 8.1 ± 8.4 and 10.1 ± 9.5, respectively, *p* = 0.12). The number of platelet transfusions received by patients in the different treatment groups was similar for the rest of the follow-up.

Hospital admission due to SAEs occurring during the study follow-up was required for 73 (52.9%) patients and was more frequent in IC and AC groups than in the patients on HMA or PC (65.9%, 63.6%, 50.9%, and 22.7%, respectively, *p* = 0.008). Nevertheless, the proportion of hospitalization days out of days under observation was similar in all treatment groups (*p* = 0.332).

Eleven patients (8.0% of patients recruited, 9.5% of those receiving active therapy) were transplanted (four autologous and seven allogeneic), ten had received IC (24.4% of patients receiving such therapy), and one had received AC (4.5% of patients receiving AC).

## 4. Discussion

The prospective observational SvLMA study described survival and HRQoL, as well as clinical characteristics and management, of patients older than 60 years treated with any of the currently available non-targeted strategies for AML in Spain (2018–2019). Our data revealed an acceptable overall patient condition and a predominantly poor MRC/LRF score, along with considerable variability between treatment groups. Median OS was 7.1 months (95% CI 4.68–9.53), and 1-year survival rate was 31.9% (95% CI 23.1–40.7). HRQoL remained stable throughout the study.

Treatment strategies ranged in our study from intensive chemotherapy (29.7%) to palliative care (15.9%) and included alternative chemotherapy (54.3%). The most common treatment option in our series was HMA (38.4%). The proportion of patients receiving IC in our study differs from other recent series [33,34] in which IC was predominant (44–54%). As regards the proportion of elderly patients with AML receiving just PC, our figure is lower than that observed in Europe (24–35%) and within the range documented in the USA (10.0–61.4%) [35]

The OS of older adults with AML reported in previous research shows wide variations as a likely consequence of differences in the studied populations and the time frame in which the treatment was administered. A population-based study carried out in the US [1] reported that 5-year OS has improved in patients aged 60–69 years from 4% in the 1980s to 24% in 2010–2017, while the improvement was very slight in those aged 70 and over (from 1% to 5% in the same time periods). The median OS of 3637 AML patients aged 60 years and over treated in Spain and reported to the PETHEMA registry between 1999 and 2013 [33] was 4.7 months, and 1-year and 5-year OS were 29% and 7%, respectively, without significant differences between treatment periods. In our study and that of PETHEMA, the median age was over 70 years (75 and 72, respectively), and the OS in the different treatment groups was short (1.0 and 1.2 months for PC; 5.9 months and 7.8 months for alternative chemotherapy; median not reached and 10.3 months for IC; respectively); 1-year OS was also similar (31.9% and 29%). HMA-treated patients in our study showed a median OS (8.8 months) similar to that obtained in a population-based study evaluating survival in decitabine and azacitidine-treated patients in the USA [36] and other European academic series [15,16].

Our data show that the MRC/LRF (which integrates cytogenetics, WBC, ECOG, age, and secondary AML) poor risk category and PC are associated with poor OS. Lee index can be used to estimate the 4-year life expectancy of any patient older than 50 years before AML diagnosis, and in our series, was associated with OS, as we also described in myelodysplastic syndromes [37], in those patients receiving active therapy. Importantly, the patients that were assigned to IC not only had a lower proportion of poor-risk MRC/LRF category than the other participating patients but also a better life expectancy prior to AML diagnosis. By contrast, we did not observe an independent association between geriatric assessment (GAH score) and OS when life expectancy was included in the model. Geriatric assessment is claimed to be a good predictor of OS in elderly AML [38], but this has only been demonstrated for some of their constitutive dimensions (i.e., cognitive function and objective physical performance) [39], and others did not find any association between physical performance and OS [40].

Fatigue score at diagnosis was not associated with OS in our patients, in contrast to what has been published in myelodysplastic syndromes [28]. The potential contribution of HRQoL for OS prediction in older adults with AML is controversial [40,41], and in our experience, EQ-5D-5L EQ-VAS at diagnosis was not associated with OS.

At present, few hematologists resort to the physician’s clinical eye (i.e., follow a gestalt approach [42]) for treatment recommendation to older adults with AML, while most use a more analytic one mainly based on age, ECOG, comorbidity burden, early death rate, etc. [12,21,24,43]. A physician’s personality and behavioral traits may also have a role in treatment assignment [44]. Our results on the Lee index score and on the selected dimensions of geriatric assessment and HRQoL evaluation agree with the published literature, showing that there is still much room for improvement in this field and that patient condition at diagnosis, including patients’ life expectancy prior to AML, should be studied thoroughly.

ED in elderly AML patients is a critical issue, and WBC is a commonly cited predictor [5,15,21,23]. In our study, we observed that WBC was significantly higher in patients who died before 8 weeks, both in the overall population and in the patients treated with HMA. The ROC analyses confirmed the validity of WBC as a predictor of ED in those populations. Nevertheless, a recent study [24] from a large cohort of patients (15–99 years old) treated with IC did not identify WBC as a predictor of ED (4 weeks). Although the new predictive scheme was also validated in a cohort of patients receiving low-intensity therapy, WBC was not directly analyzed as a predictor of ED in this patient subset. Our data suggest that the optimal cut-off may be heterogeneous for the different treatment strategies, and we also hypothesize that it might also be different for different age ranges. Even though the GAH proved reliable in assessing health status and validity to predict clinical changes [31], the results obtained in our study do not support its role as an ED predictive factor. Similarly, despite the Lee index score being independently associated with OS, it was not an appropriate predictor of ED in our series. Neither Lee index nor GAH scores were associated with TT, according to our data. Nevertheless, further research in a larger patient series should be conducted to confirm these findings before excluding the Lee index for older adults and the GAH scale as ED or TT predictors.

HRQoL evaluation is acquiring increasing importance in the decision-making process in hematologic and solid neoplasms, but there are still barriers to overcome in older adults diagnosed with AML, mainly due to the difficulty of retrieving this type of information from retrospective studies, the still anecdotal presence of HRQoL data in AML registries [45], and the lack of consensus on the best validated specific AML-HRQoL tool [46,47,48]. Fortunately, HRQoL evaluation in comparative trials on older adults with AML has become more frequent in recent times. According to our data, HRQoL improved slightly during follow-up for the overall population and, more specifically, for the HMA-treated population, but the difference was not clinically relevant since the preference values remained stable. Others have detected a functional and HRQoL decline in patients treated by IC that recovers slowly and may last 3 years or more [40,49]

Nowadays, HCT procedures have acquired a high technical level and are being used more frequently and safely in older AML adults [50]. Nevertheless, the actual proportion of older adults with AML being allotransplanted in clinical practice is extremely, small and our data (8.0%) are in agreement with other recent sources [33,51].

The SvLMA study has several limitations: (i) We have not analyzed targeted therapies because, at the outset of the study (2018), they were very infrequent in clinical practice. (ii) Genomic studies were not included in the analysis because the number of genes analyzed was very heterogeneous, and this fact precluded the use of the European LeukemiaNet prognostic scoring system. (iii) Sample size was calculated for the primary endpoints (OS and HRQoL), and consequently, the power for subgroup analysis is limited. Our study also has several strengths: its prospective and multicenter design, the comprehensive approach to the evaluation of the patient condition, and the additional evaluation of several PROs provide meaningful information about the prognosis and clinical management of older adults diagnosed with AML in Spain.

## 5. Conclusions

This real-world study showed a significant variation in overall survival between the different treatment groups. The MRC/LRF poor risk category and palliative treatment were associated with poor OS, while the MRC/LRF poor risk category and a higher Lee index score were associated with shorter OS in those patients receiving active therapy. The WBC successfully predicted early mortality in the overall population and in HMA-treated individuals. We could not predict treatment tolerability in any of the treatment groups using the GAH scale and the Lee index. HRQoL remained stable during follow-up, and the HCT rate was very low and limited to the patients treated by IC.

## Figures and Tables

**Table 1:** Patient characteristics at diagnosis.

	IC	AC	HMA	PC	Total	*p* Value
Patients receiving treatment, n (%)	41 (29.7)	22 (15.9)	53 (38.4)	22 (15.9)	138 (100.0)	
Age (years), median (IQR)	67.0 (63.8–69.8)	76.3 (73.0–81.2)	76.2 (72.0–81.5)	80.6 (78.0–86.3)	75.2 (68.6–80.6)	<0.001
Men, n (%)	19 (46.3)	13 (59.1)	33 (62.3)	14 (63.6)	79 (57.2)	0.402
BMI (kg/m^2^), mean ± SD	26.9 ± 4.2	29.9 ± 5.4	28.0 ± 5.2	25.3 ± 3.2	27.5 ± 4.9	0.012
Previous hematological disease	8 (19.5)	4 (18.2)	13 (24.5)	10 (47.6)	35 (25.5)	0.078
Lee Index score, mean ± SD	6.9 ± 2.6	9.6 ± 3.7	10.2 ± 3.3	12.5 ± 3.7	9.5 ± 3.7	<0.001
GAH scale score, mean ± SD	39.1 ± 29.3	52.2 ± 19.4	52.6 ± 23.3	51.3 ± 26.9	47.8 ± 25.8	0.195
ECOG PS, n (%)						
0–1	30 (76.9)	18 (81.8)	40 (76.9)	10 (45.5)	98 (72.6)	0.019
2–3	9 (23.1)	4 (18.2)	12 (23.1)	12 (54.5)	37 (27.4)	
EQ-VAS score, mean ± SD	67.1 ± 22.6	54.7 ± 22.5	56.5 ± 23.1	53.3 ± 27.0	58.9 ± 23.8	0.166
Marrow blast % at diagnosis, median (IQR)	38.0 (21.0–70.0)	47.5 (39.0–89.0)	35.0 (23.0–54.0)	39.0 (17.0–67.5)	39.9 (22.0–67.5)	0.219
Cytogenetics, n (%)						
Normal	24 (58.5)	15 (68.2)	29 (55.8)	14 (63.6)	82 (59.9)	
Abnormal	17 (41.5)	7 (31.8)	23 (44.2)	8 (36.4)	55 (40.1)	
Not available	0 (0.0)	0 (0.0)	1 (0.7)	0 (0.0)	1 (0.7)	
Prevalence of recurrent cytogenetic abnormalities *, n (%)						
t(8;21)	2 (4.9)	0 (0.0)	0 (0.0)	0 (0.0)	2 (1.4)	
Inv (16)	1 (2.4)	1 (14.3)	0 (0.0)	0 (0.0)	2 (1.4)	
t(9;11)	1 (2.4)	0 (0.0)	0 (0.0)	0 (0.0)	1 (0.7)	
Other **	13 (31.7)	6 (27.3)	23 (44.2)	8 (36.4)	50 (36.5)	
Patients with complex karyotype, n (%)	2 (4.9)	2 (9.0)	9 (17.3)	4 (18.2)	17 (12.4)	
MRC/LRF risk, n (%)						
Good	17 (41.5)	3 (13.6)	3 (5.7)	1 (4.5)	24 (17.4)	<0.001
Standard	16 (39.0)	6 (27.3)	13 (24.5)	4 (18.2)	39 (28.3)	
Poor	8 (19.5)	13 (59.1)	37 (69.8)	17 (77.3)	75 (54.3)	
Fatigue assessment, n (%)						
Need to stop						0.273
Not at all	12 (29.3)	7 (31.8)	6 (11.5)	1 (5.3)	26 (19.4)	
A little	13 (31.7)	7 (31.8)	20 (38.5)	8 (42.1)	48 (35.8)	
Quite a bit	7 (17.1)	5 (22.7)	14 (26.9)	7 (36.8)	33 (26.4)	
Very much	9 (22.0)	3 (13.6)	12 (23.1)	3 (15.8)	27 (20.1)	
Feeling weak						
Not at all	12 (29.3)	5 (22.7)	11 (21.2)	2 (10.0)	30 (22.2)	0.651
A little	11 (26.8)	7 (31.8)	16 (30.8)	8 (40.0)	42 (31.1)	
Quite a bit	13 (31.7)	6 (27.3)	18 (34.6)	4 (20.0)	41 (30.4)	
Very much	5 (12.2)	4 (18.2)	7 (13.5)	6 (30.0)	22 (16.3)	
Feeling tired						
Not at all	7 (17.7)	5 (22.7)	1 (1.9)	1 (5.3)	14 (10.4)	0.158
A little	15 (36.6)	6 (27.3)	22 (42.3)	9 (47.4)	52 (38.8)	
Quite a bit	10 (24.4)	7 (31.8)	16 (30.8)	3 (15.8)	36 (26.9)	
Very much	9 (22.0)	4 (18.2)	13 (25.0)	6 (31.6)	32 (23.9)	

AC, attenuated chemotherapy; BMI, body mass index; Complex cytogenetics, 3 or more abnormalities; ECOG, Eastern Cooperative Oncology Group; EQ-VAS, EuroQoL Visual Analogue Scale; GAH, Geriatric Assessment in Hematology; HMA, hypomethylating agents; IC, intensive chemotherapy; IQR, interquartile range; MRC/LRF, Medical Research Council and Leukemia Research Foundation; NA, not available; PC, palliative care; PS, performance status; SD, standard deviation. * Proportions within treatment groups are shown. ** Including complex karyotypes.

**Table 2:** Analysis of prognostic factors for overall survival (N = 138).

Overall Survival	*p* Value	HR	95% CI
Univariate analysis			
MRC/LRF risk group			
Standard/rest risk group	0.150	2.07	0.77–5.58
Unfavorable/rest risk group	0.001	4.48	1.80–11.16
Baseline EQ-VAS value	0.455	1.00	0.99–1.01
Lee scale score	<0.001	1.13	1.07–1.19
Lee ≥ 6/Lee < 6	0.004	2.33	1.31–4.15
GAH scale score	0.385	1.00	0.99–1.01
GAH > 42/GAH ≤ 42	0.899	1.04	0.59–1.81
FA score	0.392	1.00	0.99–1.01
Frontline treatment			
Palliative care/rest	<0.001	8.45	4.10–17.41
Multivariate analysis			
MRC/LRF risk group			
Unfavorable/rest risk group	<0.001	2.69	1.66–4.37
Frontline treatment			
Palliative care/rest	<0.001	4.97	2.93–8.42

CI, confidence interval; EQ-VAS, EuroQoL Visual Analogue Score; FA, fatigue assessment; GAH, Geriatric Assessment in Hematology; HR, hazard ratio.

**Table 3:** HRQoL evaluation throughout study visits.

Patient-Reported HRQoL	IC (Mean ± SD)	AC (Mean ± SD)	HMA (Mean ± SD)	Total (Mean ± SD)
EQ-VAS				
3 months	67.5 ± 17.5	75.7 ± 13.7	52.8 ± 22.1	64.0 ± 19.7
6 months	70.6 ± 18.9	78.3 ± 12.6	68.6 ± 34.0	71.0 ± 23.3
9 months	67.9 ± 16.4	70.0 ± 13.2	72.1 ± 21.6	69.5 ± 17.2
12 months	67.1 ± 19.8	81.0 ± 9.6	77.1 ± 9.9	72.2 ± 16.6
*p*-value	0.113	0.265	0.040	0.016
Preference index				
3 months	0.81 ± 0.14	0.78 ± 0.15	0.61 ± 0.32	0.75 ± 0.22
6 months	0.81 ± 0.23	0.70 ± 0.29	0.65 ± 0.45	0.74 ± 0.31
9 months	0.72 ± 0.42	0.76 ± 0.18	0.65 ± 0.29	0.71 ± 0.34
12 months	0.78 ± 0.20	0.59 ± 0.55	0.79 ± 0.24	0.74 ± 0.30
*p*-value	0.999	0.942	0.082	0.660

AC, attenuated chemotherapy; EQ-VAS, EuroQol visual Analogue scale IC, intensive chemotherapy; HMA, hypomethylating agents.

## Data Availability

Data are contained within the article and Supplementary Materials. BMS policy on data sharing may be found at https://www.bms.com/researchers-and-partners/clinical-trials-andresearch/disclosure-commitment.html.

## Supplementary Materials

The following supporting information can be downloaded at: https://www.mdpi.com/article/10.3390/jpm13121667/s1, Figure S1. Transfusion dependence at diagnosis by treatment subgroup. Figure S2. Proportion of patients alive at 12 months by treatment group. Figure S3. Proportion of patients dying before day 56 by WBC strata. Figure S4. Comparison of white blood cell counts between patients dying and surviving at 8 weeks by treatment group. Table S1. Molecular Findings at Diagnosis. Table S2. Geriatric Assessment in Hematology (GAH) Domains. Table S3. HRQoL Domains of EQ-5D-5L Questionnaire at Diagnosis. Table S4. Frontline Treatment Strategy. Table S5. Reasons for Treatment Termination by Treatment Group. Table S6. Treatment Modifications by Treatment Group.
