# Supplementary material for: Survival Outcomes and Health-Related Quality of Life in Older Adults Diagnosed with Acute Myeloid Leukemia Receiving Frontline Therapy in Daily Practice
Source: J Pers Med. 2023 Nov 28;13(12):1667. doi: 10.3390/jpm13121667 (PMC10744855; doi:10.3390/jpm13121667)
Supplement: Supplementary file 1 [file jpm-13-01667-s001.zip › jpm-2565428-supplementary.pdf]

SUPPLEMENTARY MATERIAL

Life Expectancy, Geriatric assessment, and Patient-Reported Outcomes Measurements

Life expectancy (at 4 years) before AML diagnosis was calculated with the Lee Index for Older Adults[1]. Scores range from 0-26, with a lower score corresponding to a longer life expectancy. Q1 (score <6) predicts a life expectancy at 4 years >91%, Q2 (score 6-9) 91-76%, Q3 (score 10-13) 73-52%, and Q4 (>13): 46-33%.

Geriatric assessment was performed with the aid of the Geriatric Assessment in Hematology (GAH) scale[2,3]. GAH scale evaluates patient condition using 8 items: number of drugs, walking speed, mood, difficulties for performing activities of daily life, subjective health status, nutritional status, mental status, comorbidities and smoking habit. Scores range from 0-94, with a lower score corresponding to a better patient condition.

HRQoL was measured by the EuroQoL-5L-5D questionnaire[4] and fatigue was scored (FA-score) using[5] the 3 fatigue items (No. 10, 12, and 18) of the QLQ-C30 questionnaire (i.e.: " During the past week.." Item No. 10: *"Did you need to rest?*", item No. 12 "*Have you felt weak*?", item No. 18: *"Were you tired?*"). FA score was a continuous covariate and calculated as follows: i) Raw Score= (item 1 + item 2 + item 3) / 3; ii) FA score= ((Raw Score-1) / 3) *100.

Supplementary figures

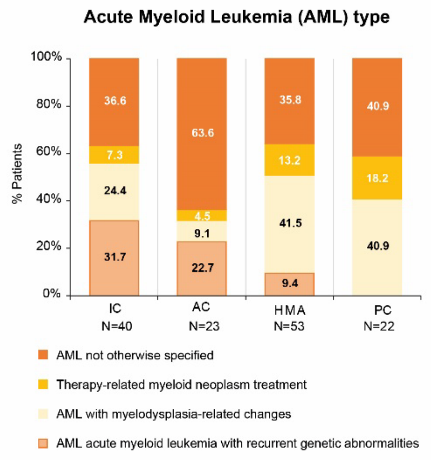

AML acute myeloid leukemia with recurrent genetic abnormalities. N (%)	IC	AC	HMA
AML with t(8;21)(q22;q22.1); RUNX1-RUNX1T1	3 (23.1)	0 (0.0)	0 (0.0)
AML with inv(16)(p13.1q22) or t(16;16)(p13.1q22);CBFB-MYH11	1 (7.7)	1 (20.0)	0 (0.0)
AML with t(9;11) (p21.3;q23.3); MLLT3-KMT2A	1 (7.7)	0 (0.0)	0 (0.0)
AML with BCR-ABL-1 (provisional entity)	1 (7.7)	0 (0.0)	0 (0.0)
AML with NPM1-mutation	7 (53.8)	2 (40.0)	5 (100.0)
AL with RUNX1-mutation (provisional entity)	0 (0.0)	2 (40.0)	0 (0.0)

AML not otherwise specified. N (%)	IC	AC	HMA	PC
AML minimally differentiated	4 (30.8)	4 (28.6)	7 (36.8)	1 (11.1)
AML without maturation	3 (23.1)	2 (14.3)	1 (5.3)	1 (11.1)
AML with maturation	3 (23.1)	4 (28.6)	3 (15.8)	3 (33.3)
Acute myelomonocytic leukemia	3 (23.1)	1 (7.1)	4 (21.1)	1 (11.1)
Acute monoblastic/monocytic leukemia	1 (7.7)	3 (21.4)	3 (15.8)	3 (33.3)
Pure erythroid leukemia	0 (0.0)	0 (0.0)	1 (5.3)	0 (0.0)

Figure S1. AML type following WHO 2016 classification criteria.

AC, attenuated chemotherapy; AML, acute myeloid leukemia; IC, intensive chemotherapy; HMA, hypomethylating agents; PC, palliative care.

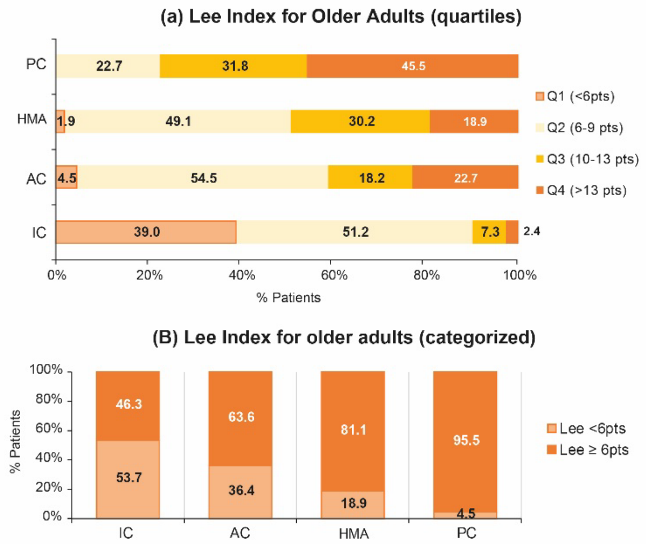

Figure S2. Lee Index for Older Adults by treatment group. (**a**) Distribution of Lee Index Score by quartiles. (**b**). Distribution of Lee Index Score by categories (score < 6 pts and score ≥6pts)

AC, attenuated chemotherapy; IC, intensive chemotherapy; HMA, hypomethylating agents; PC, palliative care.

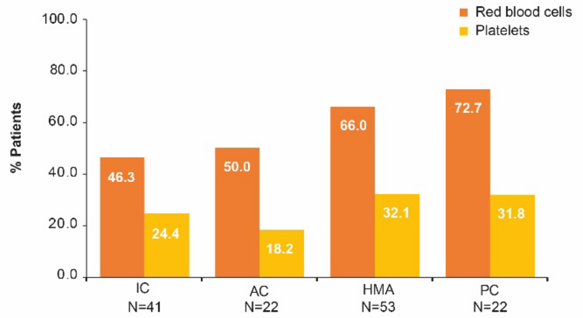

Figure S3. Transfusion dependence at diagnosis by treatment subgroup

N=138

AC, attenuated chemotherapy; IC, intensive chemotherapy; HMA, hypomethylating agents; PC, palliative care.

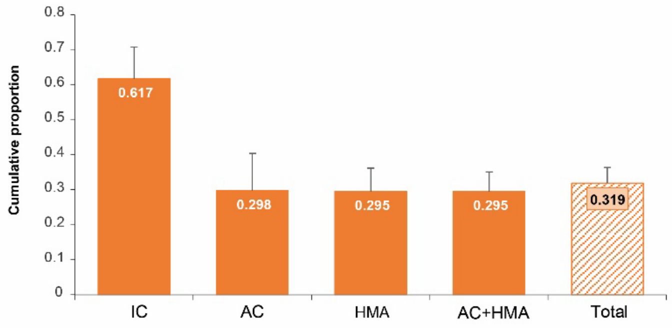

	IC	AC	HMA	AC+HMA	Total
%	61.7	29.8	29.5	29.5	31.9
IC (95%)	0.483- 0.796	0.090- 0.506	0.165-0.426	0.185 - 0.406	0.231 - 0.407

Figure S4. Proportion of patients alive at 12 months by treatment group
AC, attenuated chemotherapy; IC, intensive chemotherapy; HMA, hypomethylating agents.

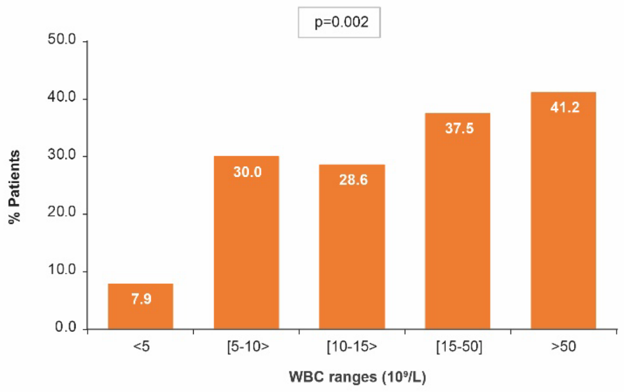

Figure S5. Proportion of patients dying before day 56 by WBC strata.

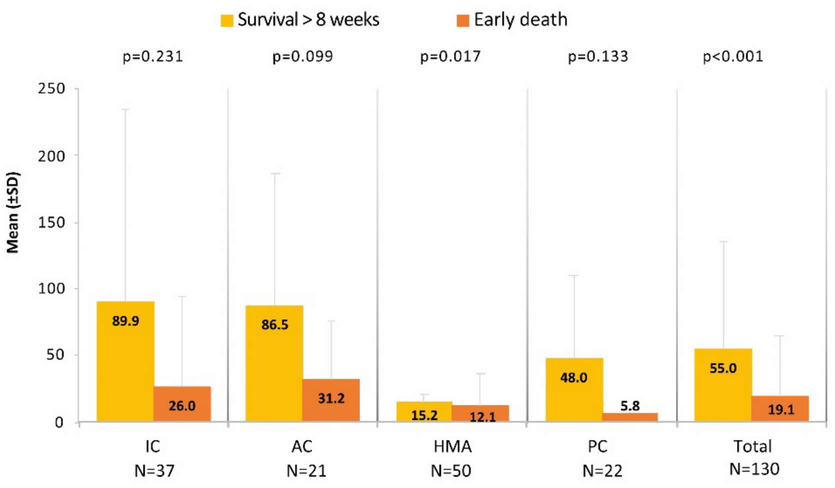

Figure S6. Comparison of white blood cell counts between patients dying and surviving at 8 weeks by treatment group.
AC, attenuated chemotherapy; IC, intensive chemotherapy; HMA, hypomethylating agents; PC, palliative care; SD, standard deviation.

Supplementary Tables

Table S1. Molecular Findings at Diagnosis

Abnormality, n (%)	IC	AC	HMA	PC	Total	Cases analyzed
*RUNX1-RUNX1T1 or RUNX1*mut	6 (20.0)	5 (27.8)	4 (18.2)	0 (0.0)	15 (19.0)	79
CBFB-MYH11	1 (3.8)	1 (6.3)	0 (0.0)	0 (0.0)	2 (2.9)	70
MLLT3-MLL	1 (5.3)	0 (0.0)	0 (0.0)	0 (0.0)	1 (1.8)	57
DEK-NUP214	0 (0.0)	0 (0.0)	0 (0.0)	0 (0.0)	0 (0.0)	40
RPN1-EVI1	1 (6.3)	1 (16.7)	1 (7.1)	1 (14.3)	4 (9.3)	43
RBM15-MLK1	0 (0.0)	0 (0.0)	0 (0.0)	0 (0.0)	0 (0.0)	36
*FLT3*mut	12 (30.0)	4 (20.0)	3 (7.7)	1 (7.1)	20 (17.7)	113
*NPM1*mut	14 (37.8)	5 (25.0)	8 (21.6)	0 (0.0)	27 (25.0)	108
*CEBPA*mut	5 (16.7)	3 (18.8)	2 (7.4)	1 (9.1)	11 (13.1)	84

AC, attenuated chemotherapy; IC, intensive chemotherapy; HMA, hypomethylating agents; PC, palliative care. The cases shown present either gene fusions or mutations.

Table S2. Geriatric Assessment in Hematology (GAH) Domains.

GAH domain, n (%)		IC	AC	HMA	PC	Total	*p* value
1: No. of drugs	<5	29 (70.7)	15 (71.4)	20 (40.0)	7 (36.8)	71 (54.2)	0.004
	≥5	12 (29.3)	6 (28.6)	30 (60.0)	12 (63.2)	60 (45.8)	
2: Gait speed	≥0.8	14 (41.2)	2 (10.5)	15 (32.6)	7 (43.8)	38 (33.0)	0.102
	<0.8	20 (58.8)	17 (89.5)	31 (67.4)	9 (56.3)	77 (67.0)	
3: Mood	Never. rarely or occasionally	27 (65.9)	16 (72.7)	36 (70.6)	11 (57.9)	90 (67.7)	0.718

	Frequently. most of the time or all time	14 (34.1)	6 (27.3)	15 (29.4)	8 (42.1)	43 (32.3)	
4: Activities of daily living	I don't need any help	32 (78.0)	15 (68.2)	29 (55.8)	7 (38.9)	83 (62.4)	0.020
	I need help in 1 or more activities	9 (22.0)	7 (31.8)	23 (44.2)	11 (61.1)	50 (37.6)	
5: Subjective health status	Excellent/Very good/good	27 (65.9)	12 (54.5)	33 (63.5)	11 (57.9)	83 (61.9)	0.811
	Fair/poor	14 (34.1)	10 (45.5)	19 (36.5)	8 (42.1)	51 (38.1)	
6: Nutrition	>8 points	18 (43.9)	8 (36.4)	14 (26.4)	6 (27.3)	46 (33.3)	0.301
	≤8 points	23 (56.1)	14 (63.6)	39 (73.6)	16 (72.7)	92 (66.7)	
7: Mental status	< 3 wrong answers	40 (97.6)	19 (95.0)	40 (90.9)	12 (75.0)	111 (91.7)	0.060
	≥ 3 wrong answers	1 (2.4)	1 (5.0)	4 (9.1)	4 (25.0)	10 (8.3)	
8: Comorbidity	0-2 points	37 (94.9)	16 (84.2)	40 (78.4)	11 (61.1)	104 (81.9)	0.013
	≥ 3 points	2 (5.1)	3 (15.8)	11 (21.6)	7 (38.9)	23 (18.1)	

AC, attenuated chemotherapy; IC, intensive chemotherapy; GAH, Geriatric Assessment in Hematology; HMA, hypomethylating agents; PC, palliative care.

Table S3. HRQoL Domains of EQ-5D-5L Questionnaire at Diagnosis

EQ-5D-5L domain, n (%)	IC	AC	HMA	PC	Total
**Mobility (N=153)**					
I have no problems in walking about	22 (53.7)	10 (45.5)	23 (43.4)	4 (21.1)	59 (43.7)
I have slight problems in walking about	11 (26.8)	7 (31.8)	11 (20.8)	8 (42.1)	37 (27.4)
I have moderate problems in walking about	5 (12.2)	3 (13.6)	10 (18.9)	3 (15.8)	21 (15.6)
I have severe problems in walking about	3 (7.3)	2 (9.1)	7 (13.2)	2 (10.5)	14 (10.4)
I am unable to walk about	0 (0.0)	0 (0.0)	2 (3.8)	2 (10.5)	4 (3.0)
**Self-care (N=135)**					
I have no problems washing or dressing myself	31 (75.6)	15 (68.2)	39 (73.6)	8 (42.1)	93 (68.9)
I have slight problems washing or dressing myself	4 (9.8)	3 (13.6)	3 (5.7)	5 (26.3)	15 (11.1)
I have moderate problems washing or dressing myself	4 (9.8)	3 (13.6)	8 (15.1)	3 (15.8)	18 (13.3)
I have severe problems washing or dressing myself	2 (4.9)	1 (4.5)	1 (1.9)	2 (10.5)	6 (4.4)
I am unable to wash or dress myself	0 (0.0)	0 (0.0)	2 (3.8)	1 (5.3)	3 (2.2)
**Usual activities** *(e.g. work. study. housework. family or leisure activities)* **(N=134)**					
I have no problems doing my usual activities	26 (63.4)	13 (59.1)	25 (48.1)	6 (31.6)	70 (52.2)
I have slight problems doing my usual activities	4 (9.8)	4 (18.2)	5 (9.6)	4 (21.1)	17 (12.7)
I have moderate problems doing my usual activities	7 (17.1)	3 (13.6)	12 (23.1)	4 (21.1)	26 (19.4)
I have severe problems doing my usual activities	3 (7.3)	1 (4.5)	5 (9.6)	1 (5.3)	10 (7.5)
I am unable to do my usual activities	1 (2.4)	1 (4.5)	5 (9.6)	4 (21.1)	11 (8.2)
**Pain/discomfort (N=135)**					
I have no pain or discomfort	18 (43.9)	9 (40.9)	17 (32.1)	9 (47.4)	53 (39.3)

I have slight pain or discomfort	14 (34.1)	5 (22.7)	17 (32.1)	7 (36.8)	43 (31.9)
I have moderate pain or discomfort	6 (14.6)	4 (18.2)	15 (28.3)	2 (10.5)	27 (20.0)
I have severe pain or discomfort	2 (4.9)	4 (18.2)	3 (5.7)	1 (5.3)	10 (7.4)
I have extreme pain or discomfort	1 (2.4)	0 (0.0)	1 (1.9)	0 (0.0)	2 (1.5)
**Anxiety/depression (N=135)**					
I am not anxious or depressed	18 (43.9)	12 (54.5)	28 (52.8)	8 (42.1)	66 (48.9)
I am slightly anxious or depressed	13 (31.7)	4 (18.2)	11 (20.8)	3 (15.8)	31 (23.0)
I am moderately anxious or depressed	6 (14.6)	4 (18.2)	9 (17.0)	4 (21.1)	23 (17.0)
I am severely anxious or depressed	3 (7.3)	1 (4.5)	4 (7.5)	3 (15.8)	11 (8.1)
I am extremely anxious or depressed	1 (2.4)	1 (4.5)	1 (1.9)	1 (5.3)	4 (3.0)

AC, attenuated chemotherapy; EQ-5D, EuroQoL-5 domains; HRQoL, health-related quality of life; IC, intensive chemotherapy; HMA, hypomethylating agents; PC, palliative care.

Table S4. Frontline Treatment Strategy

Frontline treatment strategy	n (%)
**IC combination (N=41)**	
Cytarabine (100-200 mg/m^2) + daunorubicin (60 mg/m^2)	5 (12.2)
Cytarabine (100-200 mg/m^2) + idarubicin (12 mg/m^2)	26 (63.4)
Cytarabine (100-200 mg/m^2) + idarubicin (12 mg/m^2) + midostaurin	2 (4.9)
Cytarabine (100-200 mg/m^2) + fludarabine	3 (7.3)
FLAG-IDA	1 (2.4)
FLAG-IDA + midostaurin	1 (2.4)
HDAC + idarubicin	2 (4.9)
Idarubicin + cytarabine (low dose) + dexamethasone + cyclophosphamide + vincristine	1 (2.4)
**AC combination (N=22)**	
LDAC	1 (4.5)
LDAC + cladribine	2 (9.1)
LDAC + fludarabine	9 (40.9)
FLUGA	2 (9.1)
LDAC + idarubicin	2 (9.1)
Cytarabine (100-200 mg/m^2) + idarubicin /<12 mg/m$^{2)}$	1 (4.5)
Cytarabine (100-200 mg/m^2) + daunorubicin (<45 mg/m^2)	1 (4.5)
Cytarabine (100-200 mg/m^2) + daunorubicin (<45 mg/m^2) + ATRA	1 (4.5)
Cytarabine (100-200 mg/m^2) + idarubicin (12 mg/m^2) in “5+2”schedule	3 (13.6)
HMA combination (N=53)	
Azacitidine	49 (92.5)
ATRA + Azacitidine	1 (1.9)
Decitabine	3 (5.7)
**Palliative care (N=22)**	
Etoposide	1 (4.5)
Hydroxycarbamide	4 (18.2)
Hydroxycarbamide + darbepoetin alfa	1 (4.5)
Hydroxycarbamide + etoposide	1 (4.5)
No active antineoplastic therapy	15 (68.2)

ATRA; all-trans retinoic acid; FLAG-IDA: fludarabine (30mg/m^2) + cytarabine (2g/m^2) + idarubicin (10mg/m^2) + G-CSF (granulocyte colony-stimulating factor); FLUGA, fludarabine + G-CSF (75 mg/m^2) + cytarabine; HDAC, high-dose cytarabine (1000 mg/m2 or over); LDAC, low-dose cytarabine (20 mg/m^2 or less).

Table S5. Reasons for Treatment Termination by Treatment Group

Reason for termination, N (%)	IC	AC	HMA	PC	Total
Patient's decision	0 (0.0)	1 (4.5)	2 (4.4)	0 (0.0)	3 (2.5)
Investigator's decision	7 (17.1)	8 (36.4)	5 (11.1)	0 (0.0)	20 (16.4)
Therapeutic failure	3 (7.3)	0 (0)	14 (31.1)	0 (0.0)	17 (13.9)
Death	5 (12.2)	4 (18.2)	21 (46.7)	13 (92.9)	43 (35.2)
Treatment termination	19 (46.3)	4 (18.2)	0 (0.0)	0 (0.0)	23 (18.9)
Enrollment in clinical trial	2 (4.9)	0 (0.0)	0 (0.0)	0 (0.0)	2 (1.6)
Lost to follow-up	2 (4.9)	1 (4.5)	2 (4.4)	0 (0.0)	5 (4.1)
Transplantation	1 (2.4)	1 (4.5)	1 (2.2)	0 (0.0)	3 (2.5)

Reasons for treatment termination were available in 122 patients.
AC. attenuated chemotherapy; IC. intensive chemotherapy; HMA. hypomethylating agents; PC. palliative care.

Table S6. Treatment Modifications by Treatment Group

Treatment modification type, N (%)	IC (N=7)	AC (N=11)	HMA (N=16)	PC (N=6)	Total (N=40)
Dose reduction	5 (71.4)	5 (45.5)	4 (25.0)	1 (16.7)	5 (71.4)
Suppression of any drug from the combination	0 (0.0)	1 (9.1)	0 (0.0)	0 (0.0)	0 (0.0)
Reduction in no. of cycles	3 (42.9)	2 (18.2)	0 (0.0)	0 (0.0)	3 (42.9)
Supportive treatment	0 (0.0)	2 (18.2)	4 (25.0)	3 (50.0)	0 (0.0)
Delay in cycle administration	2 (28.6)	1 (9.1)	10 (62.5)	1 (16.7)	2 (28.6)
Other	0 (0.0)	2 (18.2)	2 (12.5)	2 (33.3)	0 (0.0)

There could be more than one treatment modification. This variable was collected as a multiple response variable, and the sum of the percentages may be greater than 100%.
AC. attenuated chemotherapy; IC. intensive chemotherapy; HMA. hypomethylating agents; PC. palliative care.

References

1. Lee. S.J.; Lindquist. K.; Segal. M.R.; Covinsky. K.E. Development and validation of a prognostic index for 4-year mortality in older adults. *Jama* **2006**. *295*. 801-808. doi:10.1001/jama.295.7.801.
2. Bonanad. S.; De la Rubia. J.; Gironella. M.; Perez Persona. E.; Gonzalez. B.; Fernandez Lago. C.; Arnan. M.; Zudaire. M.; Hernandez Rivas. J.A.; Soler. A.; et al. Development and psychometric validation of a brief comprehensive health status assessment scale in older patients with hematological malignancies: The GAH Scale. *Journal of geriatric oncology* **2015**. *6*. 353-361. doi:10.1016/j.jgo.2015.03.003.
3. Cruz-Jentoft. A.J.; Gonzalez. B.; de la Rubia. J.; Hernandez Rivas. J.A.; Soler. J.A.; Fernandez Lago. C.; Arnao. M.; Gironella. M.; Perez Persona. E.; Zudaire. M.T.; et al. Further psychometric validation of the GAH scale: Responsiveness and effect size. *Journal of geriatric oncology* **2017**. *8*. 211-215. doi:10.1016/j.jgo.2016.12.008.
4. Rabin. R.; de Charro. F. EQ-5D: a measure of health status from the EuroQol Group. *Ann Med* **2001**. *33*. 337-343. doi:10.3109/07853890109002087.
5. Efficace. F.; Gaidano. G.; Breccia. M.; Voso. M.T.; Cottone. F.; Angelucci. E.; Caocci. G.; Stauder. R.; Selleslag. D.; Sprangers. M.; et al. Prognostic value of self-reported fatigue on overall survival in patients with myelodysplastic syndromes: a multicentre. prospective. observational. cohort study. *Lancet Oncol* **2015**. *16*. 1506-1514. doi:10.1016/S1470-2045(15)00206-5.
